# Integrated Solution for As(III) Contamination in Water Based on Crystalline Porous Organic Salts

**DOI:** 10.1002/advs.202403539

**Published:** 2024-06-25

**Authors:** Xiaoxia Yang, Qi Guo, Xingman Liu, Jing‐xin Ma

**Affiliations:** ^1^ State Key Laboratory of High‐Efficiency Coal Utilization and Green Chemical Engineering College of Chemistry and Chemical Engineering Ningxia University Yinchuan 750021 China

**Keywords:** adsorption, arsenic, crystalline porous organic salts, fluorescence sensing, removal

## Abstract

A stable crystalline organic porous salt (**CPOSs‐NXU‐1**) with 1D apertures has been assembled by the solvothermal method, which shows high‐sensitivity “turn‐on” fluorescence detection and large‐capacity adsorption of As(III) ions in water. The detection limits, saturated adsorption capacity, and removal rate of **CPOSs‐NXU‐1** for As(III) ions in an aqueous solution can reach 74.34 nm (5.57 ppb), 451.01 mg g^−1^, and 99.6%, respectively, at pH = 7 and room temperature. With the aid of XPS, IR, Raman, and DFT theoretical calculations, it is determined that **CPOSs‐NXU‐1** adsorbed As(III) ions in the form of H_2_AsO_3_^−^ and H_3_AsO_3_ through hydrogen bonding between the host and guest. The mechanism for fluorescence sensitization of As(III) ions to **CPOSs‐NXU‐1** is mainly to increase the energy level difference between the ground state and excited state investigated by UV–vis absorption spectra, UV–vis diffuse reflectance spectra, and theoretical calculations. By constructing fluorescent **CPOSs**, an integrated solution has been achieved to treating As(III) contamination in the water that is equipped with detection and removal. These results blaze a promising path for addressing trivalent arsenic contamination in water efficiently, rapidly, and economically.

## Graphical abstract

A fluorescent crystalline organic porous salt (**CPOSs‐NXU‐1**) is constructed, which can sensitively detect and efficiently adsorb As(III) ions in water. Through this research, an integrated material for treating As(III) contamination in water is achieved. These results show a promising path for addressing arsenic contamination in water efficiently and economically.

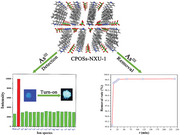

## 1 Introduction

Arsenic (As), is one of the first chemical elements known to mankind, and its compounds have been used throughout almost all of human history.^[^ ^1^ ^]^ In nature, arsenic exists primarily as arsenite, arsenate, arsenic sulfide, and a small amount of organic arsines.^[^ ^2^ ^]^ Although arsenate and organic arsine have low toxicity, they can be converted to highly toxic arsenite through biological or chemical pathways after entering water bodies, which can lead to cancerous lesions in many organs such as lung, bladder, liver, kidney, and skin cancers or acute arsenic poisoning after assimilation by the human body.^[^ ^3^, ^4^ ^]^ Consequently, the World Health Organisation (WHO) classifies arsenic compounds as one of the most threatening environmental toxic substances.^[^ ^5^ ^]^ With incomplete statistics, in the current world, at least 300 million people in developing countries have suffered from varying degrees of arsenic contamination, especially the trivalent arsenic, As(III), pollution of domestic water is particularly prominent and the situation is tending to worsen.^[^ ^4^ ^]^ WHO and the United States Environmental Protection Agency (USEPA) have stipulated that the maximum concentration of elemental arsenic in drinking water is 10 µg L^−1^ (10 ppb).^[^ ^5^ ^]^ The trace amounts of As(III) present in water are difficult to detect and remove, which makes arsenic contamination in natural water bodies a thorny ecological problem faced by the whole world.^[^ ^6^ ^]^ Currently, the following methods are commonly used for the detection of elemental arsenic: the electrochemical method,^[^ ^7^ ^]^ the colorimetric method,^[^ ^8^ ^]^ and the atomic absorption spectrometry,^[^ ^9^ ^]^ which are either cumbersome or require valuable instrumentation, and are not conducive to convenient and efficient quantitative detection. Fluorescence detection, due to its convenience, high efficiency, and low cost, has been extensively researched and applied in a wide range of fields.^[^ ^10^ ^]^ In contrast to the aforementioned detection methods, the primary benefit of using fluorescent detection for arsenic stems from the development of exceptionally selective probe molecules, enabling sample testing without pre‐treatment. Additionally, fluorescence equipment can be conducive to miniaturization and portability, facilitating true in‐field testing capabilities.^[^ ^10b^ ^]^

The detection of As is the first step to thoroughly solving the arsenic pollution in water bodies, and the efficient removal of elemental As is the crucial issue. Although various techniques exist for the removal of As from water, such as chemical precipitation, ion exchange, reverse osmosis membrane filtration, and electrodialysis, these methods generally exhibit suboptimal removal rates, high costs, and complex processes.^[^ ^11^ ^]^ Adsorptive separation is regarded as the most promising method for As treatment owing to facile operation, low cost, and reusability of adsorption materials.^[^ ^12^ ^]^ The reported materials for arsenic adsorption encompass metal oxide,^[^ ^13^ ^]^ carbon‐based materials,^[^ ^14^ ^]^ and MOFs.^[^ ^15^ ^]^ However, these adsorbent materials have significant drawbacks such as low adsorption capacity, poor selectivity, and undesirable water stability, which has emerged as a key challenge to the large‐scale application of adsorption for arsenic removal. Worse still, the vast majority of current arsenic detection and removal technologies are isolated from each other, which inadvertently increases the difficulty and cost of arsenic pollution treatment. Hence, it is of utmost scientific significance and application value to develop materials, that combine efficient and convenient detection as well as high capacity and high selectivity adsorption, to handle As(III) contamination of water bodies.

Crystalline porous organic salts (**CPOSs**), a new type of crystalline material with permanent pores formed by pairs of organic acid/base through ionic bonding,^[^ ^16^ ^]^ have been recently utilized for proton conduction,^[^ ^17^ ^]^ nonlinear mechanical properties,^[^ ^18^ ^]^ and atmospheric water harvesting^[^ ^19^ ^]^ due to their excellent stability and versatile pore structure. Comparing MOFs and **CPOSs**, it is evident that **CPOSs** have significant advantages over MOFs in terms of structural predictability and control, as well as water stability. This superiority arises from the fact that **CPOSs** are synthesized through a one‐step acid‐base neutralization reaction, rather than the multi‐step coordination substitution reactions used for MOFs. Furthermore, **CPOSs** usually have better water stability because typically prepared in water or aqueous solutions, which implies that **CPOSs** are more suitable for performance studies in aqueous systems compared to MOFs. Of course, constrained by the types and numbers of building blocks and the relatively shorter research timeline, **CPOSs** currently cannot match MOFs in terms of quantity, structural diversity, well‐defined structure‐property relationships, and scope of applications.^[^ ^20^ ^]^

Noteworthy, since the assembled blocks for constructing **CPOSs** usually consist of organic acid/base pairs with a conjugated structure, it can be predicted that by selecting appropriate organic acid/base pairs and controlling the assembly conditions, **CPOSs** with excellent fluorescence performance could be constructed for efficient fluorescence sensing of specific substances.^[^ ^21^ ^]^ At the same time, the ordered pore structure of **CPOSs** can be effectively regulated with the help of Reticular Chemistry, enabling them to efficiently adsorb specific substances.^[^ ^22^ ^]^ Therefore, it is theoretically possible to construct a new type of **CPOSs** that combines fluorescent detection and adsorptive removal to address the issue of arsenic contamination in water bodies comprehensively.

Based on the above reasons, a fluorescent **CPOSs**, [H_2_PYRIDINE ⬩ (ClO_4_)_2_]_n_ (**CPOSs‐NXU‐1**), with good chemical and physical stability was constructed by using 4,4′‐bis(pyridin‐4‐yl)biphenyl (PYRIDINE) and perchloric acid (HClO_4_) as the building blocks. The fluorescence sensing investigation showed that the **CPOSs‐NXU‐1** could efficiently detect As(III) species in water through the fluorescence “turn‐on” mechanism and remove them by efficient adsorption under appropriate conditions, accomplishing the functional integration of detection and removal. To the best of our knowledge, it is very rare that the detection and removal of As(III) species from water can be achieved simultaneously by relying on only one crystalline material.^[^ ^23^ ^]^ Versus the few materials that allow concurrent detection and separation of As ions, **CPOSs‐NXU‐1** is truly “seamless”, namely, it does not require a stepwise procedure for detection and separation by changing the valence state of arsenic.^[^ ^23^ ^]^ In addition, the mechanism of interaction between As(III) species and **CPOSs‐NXU‐1** was interpreted with the assistance of Raman, infrared (IR), X‐ray photoelectron spectroscopy (XPS), and DFT theoretical calculations.

## 2 Results and Discussion

**Figure 1:**
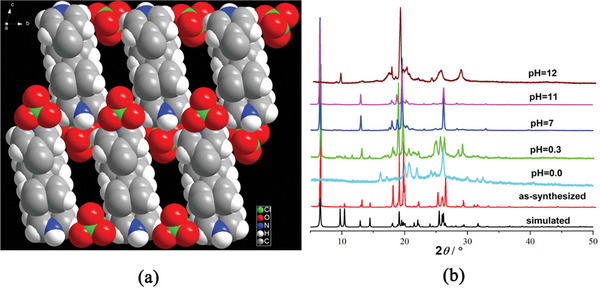
3D stacking diagram of **CPOSs‐NXU‐1** along *a*‐axis a) and PXRD pattern b).

**CPOSs‐NXU‐1** (CCDC 1 943 423) crystallizes in a triclinic crystal system with a *P*‐1 space group (Table S1, Supporting Information). The asymmetric unit consists of a doubly protonated PYRIDINE molecule (H_2_PYRIDINE^2+^) and two ClO_4_^−^ ions. Through ionic bonding, a 3D structure with 1D penetrating rectangular pores (0.77 × 0.54 nm) along the ***a***‐axis was assembled (**Figure** **1**a). Thermogravimetric analysis (TGA) demonstrated the desirable thermal stability of **CPOSs‐NXU‐1**, as depicted in Figure S1 (Supporting Information). 273 K CO_2_ adsorption experiments and NLDFT calculations revealed that the pore width of **CPOSs‐NXU‐1** ranged from 0.4 to 0.8 nm (Figure S2, Supporting Information, inset) in coincidence with the crystallographic data. Meanwhile, **CPOSs‐NXU‐1** exhibited good acid‐base stability at room temperature and could maintain structural integrity in pH = 0.3–11 aqueous solutions for at least 3 days (Figure 1b).

**Figure 2:**
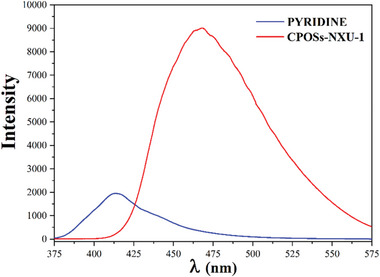
Solid‐state fluorescence spectra of PYRIDINE and **CPOSs‐NXU‐1** at room temperature.

**Figure 3:**
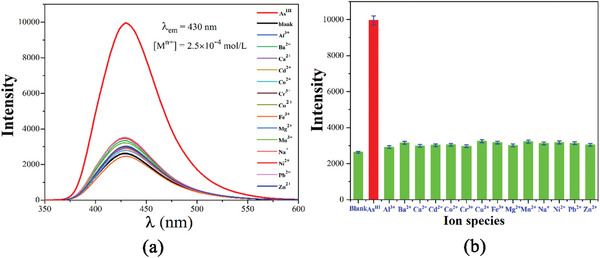
PL spectra a) and sensitization efficiency b) of **CPOSs‐NXU‐1** with different metal ions in aqueous solution.

At room temperature, **CPOSs‐NXU‐1** exhibits a bright fluorescence emission, and the maximum emission peak is located at 468 nm (with an excitation wavelength of 276 nm, **Figure** **2**), which is significantly higher than that of the free PYRIDINE ligand under the same conditions. Due to the strong luminescence emission, the fluorescence sensing ability test of **CPOSs‐NXU‐1** for different kinds of cations in water (**Figure** **3**) was carried out, in which the cation concentration was 2.5 × 10^−4^ m.

**Figure 4:**
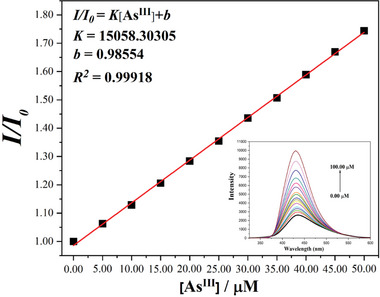
Linear relationship of **CPOSs‐NXU‐1** to As^III^ ions in water at 0.00–50.00 µm; fluorescence titration experiments (inset).

The results of fluorescence sensing showed that only As^III^ ions strongly sensitized the fluorescence emission of **CPOSs‐NXU‐1**. The fluorescence alteration caused by other tested cations was almost negligible compared to that of As^III^ (Figure 3). Meanwhile, fluorescence titration experiments (**Figure** **4**) demonstrated that the fluorescence emission intensity of **CPOSs‐NXU‐1** increased continuously with the addition of As^III^ ions (Figure 4 inset), and the relative fluorescence emission intensity (*I/I_0_*) of **CPOSs‐NXU‐1** exhibited a good linear relationship in the low to medium concentration range (0.00–50.00 µm) (Figure 4; Figure S3, Supporting Information). According to the detection limit (*DL*) formula *DL = 3δ/K* in which *δ* and *K* were the standard deviation for 30 repeated fluorescent measurements of the blank sample and the slope of the fitted equation, *I/I_0_* = *K*[As^III^]+*b*, respectively. The *DL* of **CPOSs‐NXU‐1** for As^III^ ion was 5.57 ppb (74.33 nm)^[^ ^24^ ^]^ lower than 10 ppb, the WHO requirement. The above results illustrate that **CPOSs‐NXU‐1** can be used as a reliable fluorescence sensor for detecting As(III).

**Figure 5:**
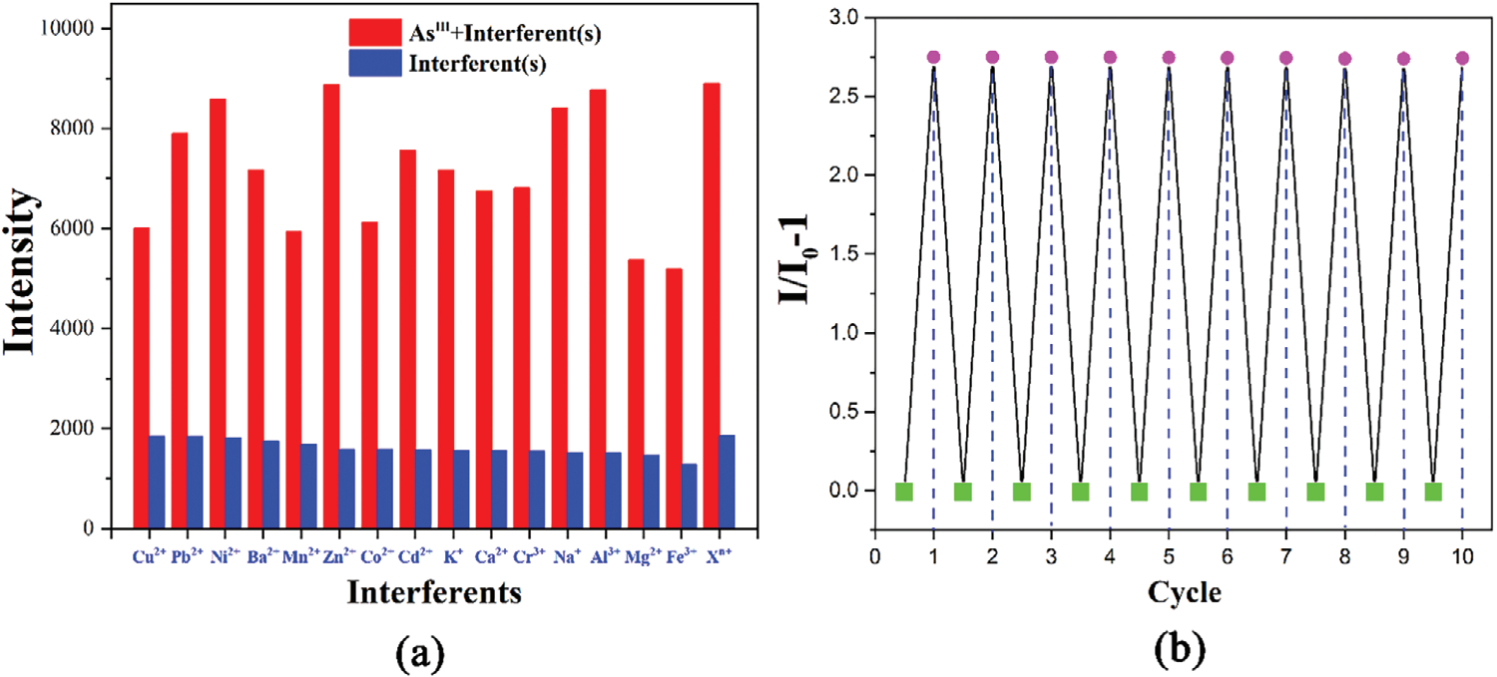
a) Fluorescence sensing of As^III^ ions by **CPOSs‐NXU‐1** in the presence of a threefold concentration of interferents. X^n+^, all the interfering ions mixed with As^III^ ions in equivalent concentrations, b) Cyclic stability of **CPOSs‐NXU‐1** sensing As^III^ ions.

Immunity to interference and recoverability are two important indicators for assessing the potential of fluorescent probe applications. Therefore, we repeated the fluorescence sensing of As^III^ ions by **CPOSs‐NXU‐1** in the presence of threefold interfering ions. The results showed that **CPOSs‐NXU‐1** still exhibited satisfactory sensitivity to As^III^ ions even when high concentrations of interfering ions were present in an aqueous solution (**Figure** **5**a). Strikingly, when we mixed all the interfering ions in equivalent concentrations with As^III^ ions (X^n+^), the matrix effect from the interferents unexpectedly vanished. The luminescence sensitivity (*I/I_0_*‐1) of **CPOSs‐NXU‐1** to As^III^ ions was almost unchanged in 10 rounds of sensing cycles (Figure 5b), and after 10 rounds of cycling, the regenerated samples of **CPOSs‐NXU‐1** showed insignificant changes in the fluorescence intensity and PXRD patterns (Figures S4 and S5, Supporting Information), as well as less than 10% mass loss of **CPOSs‐NXU‐1** after 15 rounds of cycling under the existing experimental conditions, all of which indicated that **CPOSs‐NXU‐1** could be a promising fluorescent sensor for detecting As^III^ ions in water.

**Figure 6:**
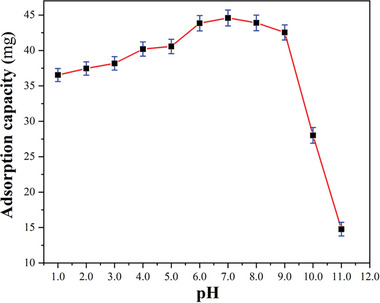
Effect of pH on adsorption of As^III^ ions by **CPOSs‐NXU‐1** at room temperature.

To completely solve the problem of trivalent arsenic contamination in water bodies, discovering the presence of the contaminant is only the first step; the key lies in efficiently removing it. Because of the presence of stable 1D pore channels in **CPOSs‐NXU‐1**, we investigated its adsorption capacity for As^III^ ions in water. Firstly, considering that the As^III^ ion has four forms in aqueous solution, H_3_AsO_3_, H_2_AsO_3_^−^, HAsO_3_^2−^, and AsO_3_^3−^, which interconverted with the change of pH. So, it is extremely necessary to investigate the effect of pH on the adsorption of As^III^ ions by **CPOSs‐NXU‐1** in the pH range where the material can be stabilized (Supporting Information). As illustrated in **Figure** **6**, the adsorption amount of **CPOSs‐NXU‐1** for As^III^ ions grew gradually with the increment of pH in the range of 1–7 at room temperature and reached the maximum value at pH = 7. However, when the solution's pH was further increased, there was a significant decrease in the adsorption amount, especially after the pH was greater than 9. Given that the framework of the material was stable in the experimental pH range, indicating that the presence of As^III^ ions in the forms of H_3_AsO_3_, H_2_AsO_3_^−^, HAsO_3_^2−^, and AsO_3_^3−^ significantly affects the adsorption capacity of the material. At pH = 7, As^III^ ions exist in an aqueous solution as H_2_AsO_3_^−^ and H_3_AsO_3_,^[^ ^25^ ^]^ so it can be presumed that **CPOSs‐NXU‐1** primarily adsorbed H_2_AsO_3_^−^ and/or H_3_AsO_3_.

**Figure 7:**
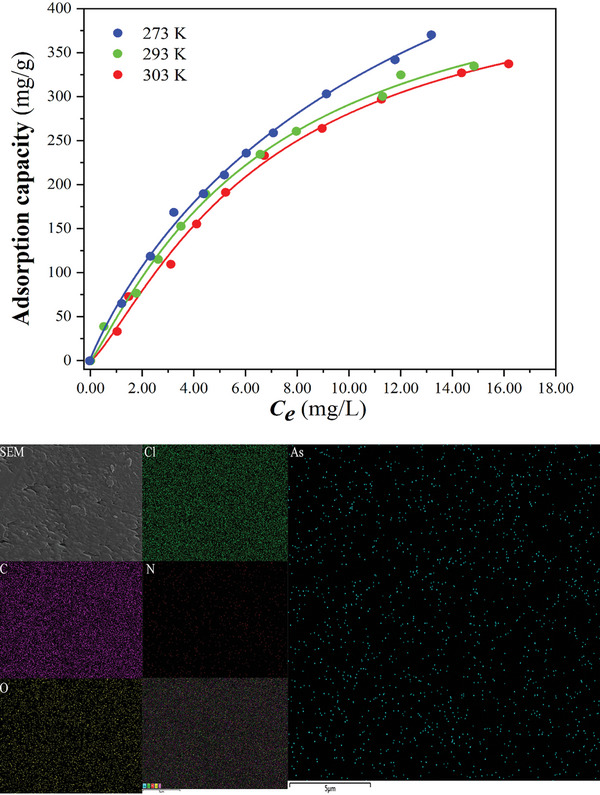
(top) Adsorption isotherms of As^III^ ions by **CPOSs‐NXU‐1** at different temperatures and (bottom) SEM‐EDS mapping of C, N, O, Cl, and As after adsorption.

Subsequently, we investigated the adsorption capacity of the materials for As^III^ ions at pH = 7. As depicted in **Figure** **7**, **CPOSs‐NXU‐1** showed excellent adsorption capacity for As^III^ ions. The maximum adsorption capacity (*Q_m_*) of **CPOSs‐NXU‐1** for As^III^ ions was calculated by fitting the Langmuir‐Freundlich‐type Baudu model^[^ ^26^ ^]^ (Equation S3 and Table S2, Supporting Information), resulting in a value of 748.99±144.99 mg g^−1^ (based on As) at 273 K, which is significantly higher than that of any other known trivalent arsenic adsorption materials to date (**Table** **1**). Although the adsorption of As^III^ ions by **CPOSs‐NXU‐1** decreases with increasing temperature, *Q*_m_ is still 451.01±37.18 mg g^−1^ at 303 K. Furthermore, SEM‐EDS mapping showed the homogeneous distribution of arsenic on **CPOSs‐NXU‐1** after adsorption of As^III^ ions (Figure 7) corroborated the effective performance of the material in adsorbing As^III^ ions.

**Table 1:** Comparison of maximum As (III) adsorption capacity of some representative materials.

Absorbent Material	*Q_m_* [mg g^−1^]	Ref.
CPOSs‐NXU‐1	748.99±144.99 (273 K) 451.01±37.18 (303 K)	This study
La_2_O_3_–CeO_2_	102.5	[27]
3Fe:2Ni:Mn	81.9	[28]
FeMnO/S(IV)	40.5	[29]
Fe─Cu─Mn (ICM)	131.0	[30]
Fe/Mn‐MOFs	69.17	[31]
Nanohydrated ZrO_2_/GO	95.0	[32]
Fe‐Co@MOF‐74	266.52	[33]
NH_2_‐MIL‐101(Fe)	153.4	[34]
UiO‐66@PGC20%	270.58	[35]
Zn‐MOF‐74	211	[36]
β‐MnO_2_@ZIF‐8	140.47	[37]
δ‐MnO_2_@Fe/Co‐MOF‐74	300.5	[38]
Zr/Mn/C	132.28	[39]
MnO_2_@La(OH)_3_	139.9	[40]
CeMn@9CNTs	151.06	[41]

**Figure 8:**
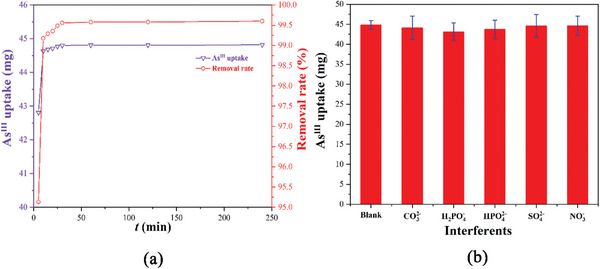
a) The relationship between time and adsorption as well as removal rate of **CPOSs‐NXU‐1** on As^III^ ions and b) adsorption selectivity in the presence of anionic impurities.

The ability to quickly and efficiently remove As^III^ ions from the water is another key factor that determines whether the material can finally be applied in practical applications. Based on the above experimental results, the adsorption kinetics of **CPOSs‐NXU‐1** on As^III^ ions were investigated at room temperature, and the experimental conditions and procedures were detailed in Supporting Information. As shown in **Figure** **8**a, **CPOSs‐NXU‐1** adsorbed up to 44.6 mg of As^III^ ions within 10 min in an aqueous solution with a total trivalent‐arsenic content of 45.0 mg and pH = 7, giving a removal rate of 99.1%, while the adsorption of 44.8 mg in 30 min corresponded to a clearance of 99.6%. Continuing to extend the adsorption time, it can be seen that the adsorption amount and removal rate of As^III^ ions by **CPOSs‐NXU‐1** almost maintained the constant, implying that the adsorption of As^III^ ions by the material has reached equilibrium. We chose the quasi‐primary kinetic equation (Equation S4, Supporting Information) and quasi‐secondary kinetic equation (Equation S5, Supporting Information) to fit the adsorption data (Figure S6 and Table S3, Supporting Information). The results showed that the adsorption of As^III^ ions by **CPOSs‐NXU‐1** was more suitable for quasi‐secondary kinetic adsorption (*R^2^* > 0.9999), implying the presence of definite As^III^ ion adsorption sites.^[^ ^42^ ^]^ Considering the practical application scenarios, we introduced an equal mass of anionic impurities (CO_3_^2−^, H_2_PO_4_^−^, HPO_4_^2−,^ NO_3_^−^, and SO_4_^2−^) into the above experimental system. This allowed us to investigate the adsorption selectivity of **CPOSs‐NXU‐1** for As^III^ ions in natural water (Figure 8b). The results showed that in the presence of an equal mass of anionic impurities and with an adsorption time of 30 minutes, the amount of As^III^ ions adsorbed by **CPOSs‐NXU‐1** were all greater than 43.0 mg (removal rate >95.5%), indicating a good adsorption selectivity for As^III^ ions. The high removal rate, rapid attainment of adsorption equilibrium, and specific adsorption selectivity demonstrate that **CPOSs‐NXU‐1** can effectively remove As^III^ ions from water.

**Figure 9:**
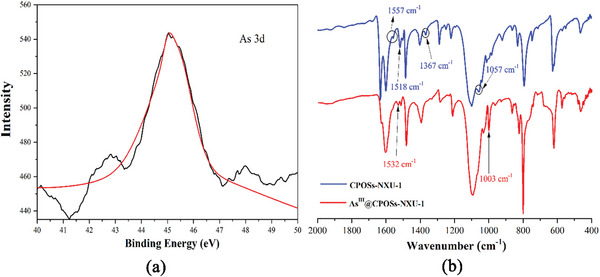
a) XPS spectrum of **As^III^@ CPOSs‐NXU‐1**, b) IR of **CPOSs‐NXU‐1** and **As^III^@ CPOSs‐NXU‐1**.

**Figure 10:**
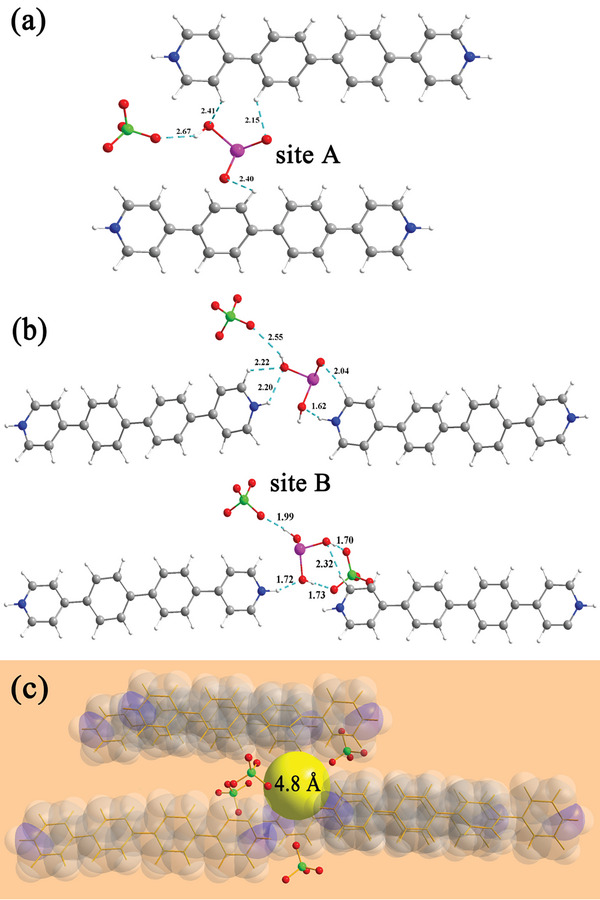
Adsorption sites and interactions between **CPOSs‐NXU‐1** and HAsO_3_^2−^ a) and H_2_AsO_3_^−^/H_3_AsO_3_ b), the cavity near site B with an approximate diameter of 4.8 Å c). Color code: magenta, As; red, O; gray, C; white, H; blue, N; green, Cl; yellow, the cavity schematic sphere.

To clarify what kind of forms of As^III^ ions are adsorbed by **CPOSs‐NXU‐1** and to better elucidate the adsorption mechanism, we initially investigated the XPS, IR, and Raman spectra of the sample after the adsorption process, **As^III^@ CPOSs‐NXU‐1** (**Figures** **9** and **10**; Figures S7–S9, Supporting Information). The XPS results showed that the characteristic peak at 44.9 eV (Figure 9a) which indicated the adsorbed arsenic in **CPOSs‐NXU‐1** was indeed a trivalent species.^[^ ^43^ ^]^ Additionally, the peaks at 532.4, 399.8, 285.3, and 208.3 eV corresponded to O1s, N1s, C1s, and Cl2p respectively (Figure S7, Supporting Information). The IR spectra (Figure 9b) demonstrated that the peak at 1003 cm^−1^ exhibited an antisymmetric telescoping vibrational of As‐OH,^[^ ^44^ ^]^ while the corresponding Raman spectrum (Figure S8, Supporting Information) displayed *C_S_* symmetric and asymmetric stretching vibrations of As‐OH at 543 and 642 cm^−1^, also, As‐O stretching vibrations at 766, 805, and 851 cm^−1^ are observed in the Raman spectra, all of which imply that the adsorbed substances may be H_2_AsO_3_^−^ and/or H_3_AsO_3_.^[^ ^45^ ^]^ Comparing the IR spectra of **CPOSs‐NXU‐1** and **As^III^@CPOSs‐NXU‐1**, it can be observed that the peaks at 1367 and 1057 cm^−1^ in **CPOSs‐NXU‐1**, which were attributed to the in‐plane and out‐of‐plane rocking vibrational of Cl─O,^[^ ^46^ ^]^ had disappeared in **As^III^@CPOSs‐NXU‐1** suggested an interaction between ClO_4_^−^ and adsorbed H_2_AsO_3_^−^ or H_3_AsO_3_, indicating that ClO_4_^−^ is the site of adsorption. Meanwhile, the double strong peaks at 1659–1564 cm^−1^ and the weak peak at 1557 cm^−1^ in **CPOSs‐NXU‐1** can be assigned to the N─H vibration of pyridinium inner salts,^[^ ^47^ ^]^ while in **As^III^@CPOSs‐NXU‐1** those become single and disappears respectively, and the small peak that used to be at 1518 cm^−1^ was displaced to 1532 cm^−1^ and weakened in intensity, pointing the N─H vibration of the pyridinium inner salt is restricted after adsorption and suggests that the pyridinium inner salt might serve as another adsorption site. In addition, the peaks of **As^III^@ CPOSs‐NXU‐1** in the range of 3400–2600 cm^−1^ were significantly weakened or even disappeared concerning those of **CPOSs‐NXU‐1** (Figure S9, Supporting Information) meaning that the original C─H and N─H vibrations in **CPOSs‐NXU‐1** were suppressed by adsorbed H_2_AsO_3_^−^ and/or H_3_AsO_3_ through host‐guest interactions.^[^ ^48^ ^]^

**Figure 11:**
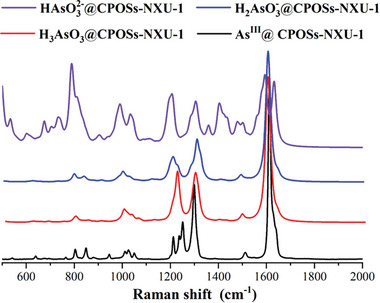
Calculated **HAsO_3_^2−^/H_2_AsO_3_^−^/H_3_AsO_3_@CPOSs‐NXU‐1** and measured Raman spectra of **As^III^@CPOSs‐NXU‐1** at room temperature.

To further confirm the aforementioned speculations, we conducted theoretical calculations (Supporting Information) on the adsorption of As^III^ ions by **CPOSs‐NXU‐1** using Density Functional Theory (DFT), with HAsO_3_^2−^, H_2_AsO_3_^−^, and H_3_AsO_3_ as adsorbates, respectively. As shown in Figure 10, there are two adsorption sites, A and B, in **CPOSs‐NXU‐1**. Site A is located at the center of the 1D pore channel of **CPOSs‐NXU‐1** and adsorbs HAsO_3_^2−^ through the hydrogen bonds formed by the O atoms in ClO_4_^−^ and the H atoms on the aryl ring of H_2_PYRIDINE^2+^ with the H and O atoms of HAsO_3_^2−^ (Figure 10a). Site B is positioned near the pyridinium inner salt of **CPOSs‐NXU‐1**, which adsorbs H_2_AsO_3_^−^/H_3_AsO_3_ through the formation of hydrogen bonds between the O atoms in ClO_4_^−^ and the H atoms of the aryl ring of H_2_PYRIDINE^2+^ and the pyridinium inner salt with the H and O atoms of the adsorbent (Figure 10b). Calculations of the heat of adsorption (*E_ads_*) revealed that the *E_ads_* values for the adsorption of HAsO_3_^2−^ and H_2_AsO_3_^−^/H_3_AsO_3_ at sites A and B were 1.05 and −1.71/−1.36 eV, respectively. Considering that the heat of adsorption represents an exothermic process, it can be concluded that the absorption of HAsO_3_^2−^ is not very convincing. One step further, we calculated the Raman spectra of **CPOSs‐NXU‐1** after the adsorption of HAsO_3_^2−^, H_2_AsO_3_^−^, and H_3_AsO_3_, the calculated spectra of **H_2_AsO_3_^−^@CPOSs‐NXU‐1** and **H_3_AsO_3_@ CPOSs‐NXU‐1** are in high coincidence with the measured **As^III^@ CPOSs‐NXU‐1** spectra (**Figure** **11**). In stark contrast, the spectrum of **HAsO_3_^2−^@CPOSs‐NXU‐1** is distinctly different from the measured ones. A combination of experimental and theoretical calculations confirms that the arsenic species adsorbed by **CPOSs‐NXU‐1** are H_2_AsO_3_^−^ and H_3_AsO_3_ and the adsorption site is located near the pyridinium inner salt. With the identification of adsorption sites and adsorbate species, the problem of cation adsorption selectivity is then fully explained. Under the experimental conditions, all cations except As^III^ ions exist as positively charged hydrated ions, [M(H_2_O)_x_]^n+^, however, the pyridinium inner salt, which plays a key role in the adsorption, is also electropositive, which means [M(H_2_O)_x_]^n+^ not being able to approach the pyridinium inner salt due to electrostatic repulsion. On the contrary, the H_2_AsO_3_^−^ and H_3_AsO_3_ can easily approach the pyridinium inner salt by electrostatic attraction and form hydrogen bonds to enhance interaction. Moreover, the diameter of the cavity surrounding site B is ≈4.8 Å matched to the diameters of H_2_AsO_3_^−^ and H_3_AsO_3_ (∼4.69 Å, based on geometry optimization, Supporting Information), rather markedly larger than the anions commonly found in the water, e.g., H_2_PO_4_^−^ (3.73–4.42 Å), HPO_4_^2‐^(3.96–4.50 Å), NO_3_^−^ (2.37–3.28 Å), SO_4_^2−^ (2.90–3.40 Å), and CO_3_^2−^ (2.50–2.74 Å),^[^ ^49^ ^]^ which indicated that the geometric size matching was another main reason for the high adsorption selectivity of H_2_AsO_3_^−^ and H_3_AsO_3_ by **CPOSs‐NXU‐1**.

**Figure 12:**
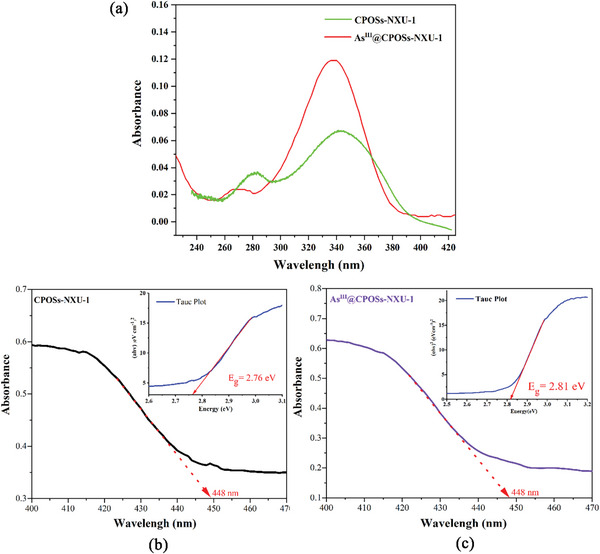
UV–vis absorption a) and diffuse reflectance spectra b,c) of **CPOSs‐NXU‐1** and **As^III^@ CPOSs‐NXU‐1**.

To elaborate the fluorescence sensitization mechanism of **CPOSs‐NXU‐1** by H_2_AsO_3_^−^ and H_3_AsO_3_, we examined the PXRD (Figure S5, Supporting Information), UV–Vis absorption spectra (**Figure** **12**a), and diffuse reflectance spectra (Figure 12b,c) before and after adsorption. As shown in Figure S5 (Supporting Information), the PXRD spectra of **CPOSs‐NXU‐1** had not changed before and after adsorption, suggesting that the framework of **CPOSs‐NXU‐1** remained intact during the adsorption process. After adsorbed H_2_AsO_3_^−^ and H_3_AsO_3_, the thermal vibrations of N─H, Cl─O, C─H, and O─H of **CPOSs‐NXU‐1** in the excited state could be dramatically restricted through host‐guest interactions (Figure 10b), which enhanced the luminescence intensity of **CPOSs‐NXU‐1**.^[^ ^50^ ^]^

Meanwhile, UV–vis absorption spectra showed that the absorption peak of **CPOSs‐NXU‐1** at 342 nm was blueshifted to 336 nm after adsorbed H_2_AsO_3_^−^ and H_3_AsO_3_, implying that the energy difference between the ground state and the excited state of the host was increased by adsorbed trivalent arsenic species. The bandgap widths of **CPOSs‐NXU‐1** and **As^III^@CPOSs‐NXU‐1** are 2.76 and 2.81 eV, respectively, determined using the empirical formula (Equation S7, Supporting Information) through UV–vis diffuse reflectance spectroscopy which represented the adsorbed H_2_AsO_3_^−^ and H_3_AsO_3_ could increase the material's bandgap and electron transition energy, which in turn might enhance fluorescence emission.^[^ ^51^ ^]^

**Figure 13:**
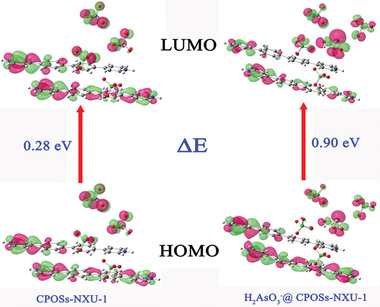
LUMO and HOMO of **CPOSs‐NXU‐1** and **H_2_AsO_3_^−^@ CPOSs‐NXU‐1**.

Simultaneously, the LUMO and HOMO energies of **CPOSs‐NXU‐1** and **H_2_AsO_3_^−^@CPOSs‐NXU‐1** were calculated (**Figure** **13**) and the results showed that the energy difference between the LUMO and HOMO of the materials increased from 0.28 to 0.90 eV after adsorbed H_2_AsO_3_^−^. The widening of the energy level difference led to a stronger fluorescence emission.^[^ ^52^ ^]^

## 3 Application in Real Water

**Figure 14:**
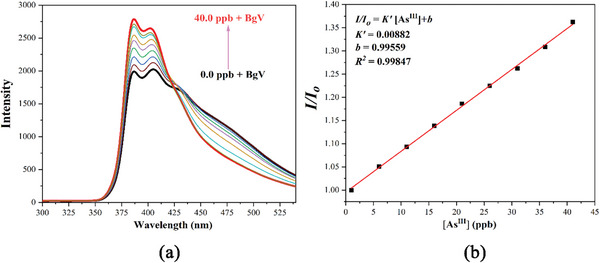
The fluorescence titration experiments a) and linear relationship b) of **CPOSs‐NXU‐1** to As^III^ ions in real water at 0.0 + BgV to 40.0 + BgV ppb.

To verify the fluorescence sensing and removal ability of **CPOSs‐NXU‐1** on As^III^ ions in real water, we took the surface runoff water of the Yellow River in Yinchuan as a sample for the experiment. First, the water samples were filtered with a 0.2 µm membrane to remove the insoluble matter visible to the naked eye. Second, the As content in the water samples was measured by ICP‐MS, and the result was 0.997 ppb (Background Value, BgV), which was lower than the *DL* of **CPOSs‐NXU‐1** for As^III^ ions. So, **CPOSs‐NXU‐1** could not be used for direct fluorescence sensing of the taken water samples. To fix that, we repeated the fluorescence titration experiment of **CPOSs‐NXU‐1** to As^III^ ions with filtered Yellow River water replacing distilled water. A small amount of hydroxylamine hydrochloride was added to the filtered Yellow River water before the experiment started to ensure the As was in the trivalent form. The experiments showed that the fluorescence emission of **CPOSs‐NXU‐1** increased significantly with As^III^ ions concentration in the range of 0.0 ppb + BgV to 40.0 ppb + BgV (5 ppb concentration interval), and the linear relationship between *I/I_0_* and As^III^ ions concentration remained good (**Figure** **14**). The change in the shape of the emission peaks should be attributed to the interaction between **CPOSs‐NXU‐1** and some substances in the Yellow River water. Besides, 20.0 mg of **CPOSs‐NXU‐1** was added into 50.0 mL of filtered Yellow River water and stirred for 30 min at room temperature, followed by centrifugation. The As content in the supernatant was 0.047 ± 0.002 ppb measured by ICP‐MS, corresponding to a removal rate of 95.3%, which indicates that **CPOSs‐NXU‐1** has a desirable efficacy in removing arsenic from the real water samples.

## 4 Conclusion

In this work, we have constructed a new structurally stable fluorescent crystalline organic porous salt material, **CPOSs‐NXU‐1**. It has been demonstrated that this material not only can achieve highly sensitive fluorescence detection of As^III^ ions in water but also exhibits excellent adsorption capacity for As^III^ ions. The detection limits, maximum adsorption capacity, and removal rate of **CPOSs‐NXU‐1** can reach 5.57 ppb, 451.01 mg/g, and 99.6% in an aqueous solution at pH = 7 and room temperature with satisfactory recyclability. Through the utilization of XPS, IR, Raman, and DFT theoretical calculations, the form of the adsorbed As^III^ ions, the adsorption sites, and the type of adsorption driving force have been identified. The main mechanism for fluorescence sensitization of **CPOSs‐NXU‐1** by As^III^ ions is clarified by UV–Vis absorption spectra, UV–Vis diffuse reflectance spectra, and theoretical calculations. By constructing **CPOSs** with excellent fluorescence performance, we achieved the integrated treatment of As(III) pollutants in water which provided a new and efficient approach to solving arsenic pollution economically. These results suggest that when constructing fluorescent **CPOSs** to meet different needs, detection, and/or adsorption, we should first design the active sites based on the specific form of the target and the potential types of interactions, then, determine the suitable acid‐base pairs in combination with the spatial volume of the target. Finally, continuous experimentation is necessary until the optimal solution is found. Relevant work is currently progressing in an orderly manner.

## 5 Experimental Section

### Synthesize CPOSs‐NXU‐1

4, 4′‐Bis(pyridin‐4‐yl)biphenyl (PYRIDINE, 12 mg, 0.04 mmol), 6 m HClO_4_ (2 mL)_,_ and H_2_O (14 mL) were stirred for 1 h at room temperature then transferred the mixture into a 25 mL polytetrafluoro reactor. The reaction kettle was placed in a constant temperature oven at 160 °C for 3 days and then cooled to room temperature to obtain needle‐like transparent crystals. These crystals were filtered, washed three times with 10 mL of distilled water, and naturally dried in air to obtain **CPOSs‐NXU‐1** (yield: 75%, based on PYRIDINE).

### Luminescence Sensing Measurements

The fine ground **CPOSs‐NXU‐1** (50.0 mg) was dispersed in 50 mL water and treated by ultrasonication for approximately 60 min, and then aged for 24 h to form stable suspensions. The obtained suspension was used for fluorescent sensing experiments. For cations luminescence detection, 50 µL of 10.0 mmol L^−1^ M(NO_3_)_n_ (n = 1–3) (M = Na^+^, Mg^2+^, Ca^2+^, Cu^2+^, Pb^2+^, Cd^2+^, Co^2+^, Ni^2+^, Zn^2+^, Mn^2+^, Ba^2+^, Al^3+^, Cr^3+^, and Fe^3+^) and As(III) ion aqueous solution was added to a 1.95 mL suspension.

### Regeneration of CPOSs‐NXU‐1

Post‐experimental **CPOSs‐NXU‐1** was collected by centrifugation, washing with deionized water, filtration, and vacuum drying for 8 h at 60 °C. The collected sample was stirred in pH = 11 NaOH solution for 1 h, then centrifuged and washed, the same process was repeated three times. Further, the sample was immersed in 0.01 m HClO_4_ solution overnight, followed by centrifugation, washing with deionized water, washing with anhydrous alcohol, and vacuum drying for 12 h at 50 °C. Ultimately, the regenerated **CPOSs‐NXU‐1** were obtained.

## Conflict of Interest

The authors declare no conflict of interest.

## Supporting information

Supporting Information

Supporting Information

## Data Availability

The data that support the findings of this study are available from the corresponding author upon reasonable request.
