# Supplementary material for: Integrated Solution for As(III) Contamination in Water Based on Crystalline Porous Organic Salts
Source: Adv Sci (Weinh). 2024 Jun 25;11(32):2403539. doi: 10.1002/advs.202403539 (PMC11348186; doi:10.1002/advs.202403539)
Supplement: Supplementary file 1 — Supporting Information [file ADVS-11-2403539-s002.docx]

Supporting Information

**Integrated Solution for As(III) Contamination in Water Based on Crystalline Porous Organic Salts**

Xiaoxia Yang, Qi Guo, Xingman Liu, and Jing-xin Ma*

**Materials**

All reagents and solvents were commercially available and used without further purification. Distilled water is produced by a laboratory water purification system. A 5 mg/mL trivalent arsenic solution was prepared by dissolving As_2_O_3_ (99.995%,) in 37% HCl under adequate protection, then diluted as required.

***Caution!*** Perchloric acid with organic ligands is potentially explosive, trivalent arsenic solution and As_2_O_3_ are highly toxic. Only a small amount should be used, and special attention should be paid when necessary. The complex and experimental procedure described in this report has, so far, been found to be safe when used in small quantities.

**Characterization**

Single-crystal X-ray diffraction and crystal resolution data were collected at 150.15 K using a Bruker APEX-II CCD diffractometer with graphite monochromatic Cu *Kα* radiation (λ = 1.54184Å). Structures were resolved using the ***SHELX-2014*** program through direct methods and refined on *F^2^* utilizing full matrix least squares. Anisotropy was applied to all non-hydrogen atoms, while hydrogen atoms were treated with isotropy. FT-IR spectra were measured using a Fourier Transform Infrared Spectrometer (FTIR S2) with KBr presses, covering a spectral range of 4000-400 cm^-1^. Thermogravimetric analysis was conducted on a SETARAM SETSYS16 Thermal Analyser under nitrogen atmosphere from room temperature to 800 °C at a ramping rate of 10 °C/min. Powder X-ray diffraction data were collected on a Rigaku SmartLab SE diffractometer with a scanning step of 0.02° and a *2θ* range of 5-50°. CO_2_ adsorption isotherms were measured using a Micromeritics ASAP 2460 surface area analyzer. As(III) ion adsorption experiments were performed isothermally on a model ZWY-240 constant temperature incubation oscillator. X-ray photoelectron spectroscopy (XPS) was performed using a Thermo Escalab 250Xi spectrometer equipped with an Al *Ka* source. As(III) ion concentration determination were collected on a NexION 350X Inductively Coupled Plasma Mass Spectrometer (ICP-MS). Raman spectra of the samples were analyzed using a Thermo Fisher DXR Laser Raman Spectrometer with 780 nm single longitudinal mode laser. Fluorescence determination was performed using a Hitachi F-7100 spectrofluorimeter equipped with *in situ* electromagnetic stirrer. UV-Vis absorption spectra were measured on a TU-1901 dual-beam UV-Vis spectrophotometer.

**As(III) Adsorption Experiment**

**pH effect on arsenic adsorption capacity**

At room temperature, 10.0 mg of **CPOSs-NXU-1** was added to 9.0 mL of 5.0 mg/mL As(III) ion solution, and the solution pH range was set from 1.0 to 11.0 with 0.2 M HCl and/or NaOH and the final volume was controlled to not exceed 30 mL, then, the mixture was shaken at 180 rpm for 1 hour. The supernatant was rapidly filtered through a dry and clean glass tube fitted with a 0.45 μm membrane filter. The filtered solution was then analyzed by ICP-MS to measure adsorbed As(III) content.

**As(III) isotherm adsorption**

The equilibrium adsorption isotherm was investigated by adding 10.0 mg of the as-synthesized **CPOSs-NXU-1** samples to 40.0 mL of trivalent arsenate solution with different initial concentrations (10.0∼100.0 mg/L) at pH = 7.0. The suspensions were then shaken in a thermostatic incubator shaker for 4 h at 273, 293, and 303 K respectively to reach adsorption equilibrium. The residual arsenate concentration was analyzed by ICP-MS. Arsenic adsorption and removal rate were calculated by the following equation（Eq. S1 and S2）, in which *Q_e_*, *C_0_*, *C_e_*, V, and m are the equilibrium adsorption capacity (mg/g) of As(III) by **CPOSs-NXU-1**, the starting and equilibrium concentration (mg/L), the volume of trivalent arsenate solution (L), and the dry weight (g) of **CPOSs-NXU-1**, respectively. The Langmuir-Freundlich-type Baudu model (Eq. S3) was used to fit experimental data.

$Q_{e}=\frac{(C_{0}-C_{e})V}{m}$ (Eq. S1)

$\mathrm{Re}moval\text{ }Rate\text{ = }\frac{C_{0}-C_{e}}{C_{0}}\times100\%$ (Eq. S2)

$Q_{e}=Q_{m}\times\frac{KC_{e}^{(1+n)}}{1+KC_{e}^{(1+n)}}$ (Eq. S3)

in which *Q_m_* and *K* are the maximum adsorption capacity (mg/g) and the adsorption equilibrium constant (L/mg) reflecting the affinity between the adsorbent and the adsorbate, respectively.

**Kinetic experiment**

At 293 K, add 9 mL of As(III) solution with a concentration of 5.0 mg/mL to a 50 mL volumetric flask, and set the solution pH at 7.0 with 0.1 M HCl and/or NaOH, the final volume was controlled to not exceed 30 mL. Then, 10.0 mg **CPOSs-NXU-1** was added, and the mixture was shaken in a constant temperature shaker at a velocity of 180 rpm. At the given time intervals, the suspensions were withdrawn and then filtrated through a 0.45 μm filter membrane immediately. The concentration of the residual arsenate species in the filtrates was determined by ICP-MS measurements. Pseudo-first order kinetic model (Eq. S4) and Pseudo-second-order kinetics model (Eq. S5) are defined as:

$ln\left(Q_{e}-Q_{t}\right)=lnQ_{e}-k_{1}\times t$ (Eq. S4)

$\frac{t}{Q_{t}}=\frac{1}{k_{2}\times Q_{e}^{2}}+\frac{t}{Q_{e}}$ (Eq. S5)

Where *Q_t_* (mg/g) is the adsorbed ion per gram of adsorbent at each time (*t*, min), *k_1_* is the adsorption constant (1/min), and *k_2_* is second-order kinetics rate constant (g/(mg🞘min)). The fitting results are shown in Table S3.

**Density Functional Theory (DFT) Calculations**

The DFT calculation was carried out using the CASTEP package. A OTFG norm conserving pseudopotentials was selected. The general gradient approximation with the Perdew-Burke-Ernzerh function (GGA-PBE) was applied for the electronic structure. The TS custom method for DFT-D was used. The convergence tolerances of energy, maximum force, maximum stress, and maximum displacement for structural optimization were 1.0 × 10^-5^ eV/atom, 0.03 eV/Å, 0.05 GPa, and 0.001 Å, respectively. The self-consistent field (SCF) density convergence tolerance was 1×10^-6^. The adsorption energy (*E_ads._*) of the A and B was defined as：

*E_ads._* = *E*(A/B) – *E*(A) – *E*(B) (Eq. S6)

where *E*(A/B), *E*(A), and *E*(B) were the energy of A adsorbed with B, the energy of A, and the energy of B, respectively.

**Calculation of the bandgap widths**

Empirical formulas:

$E_{g}=\frac{1240}{\lambda}$ (Eq. S7)

**Figures:**


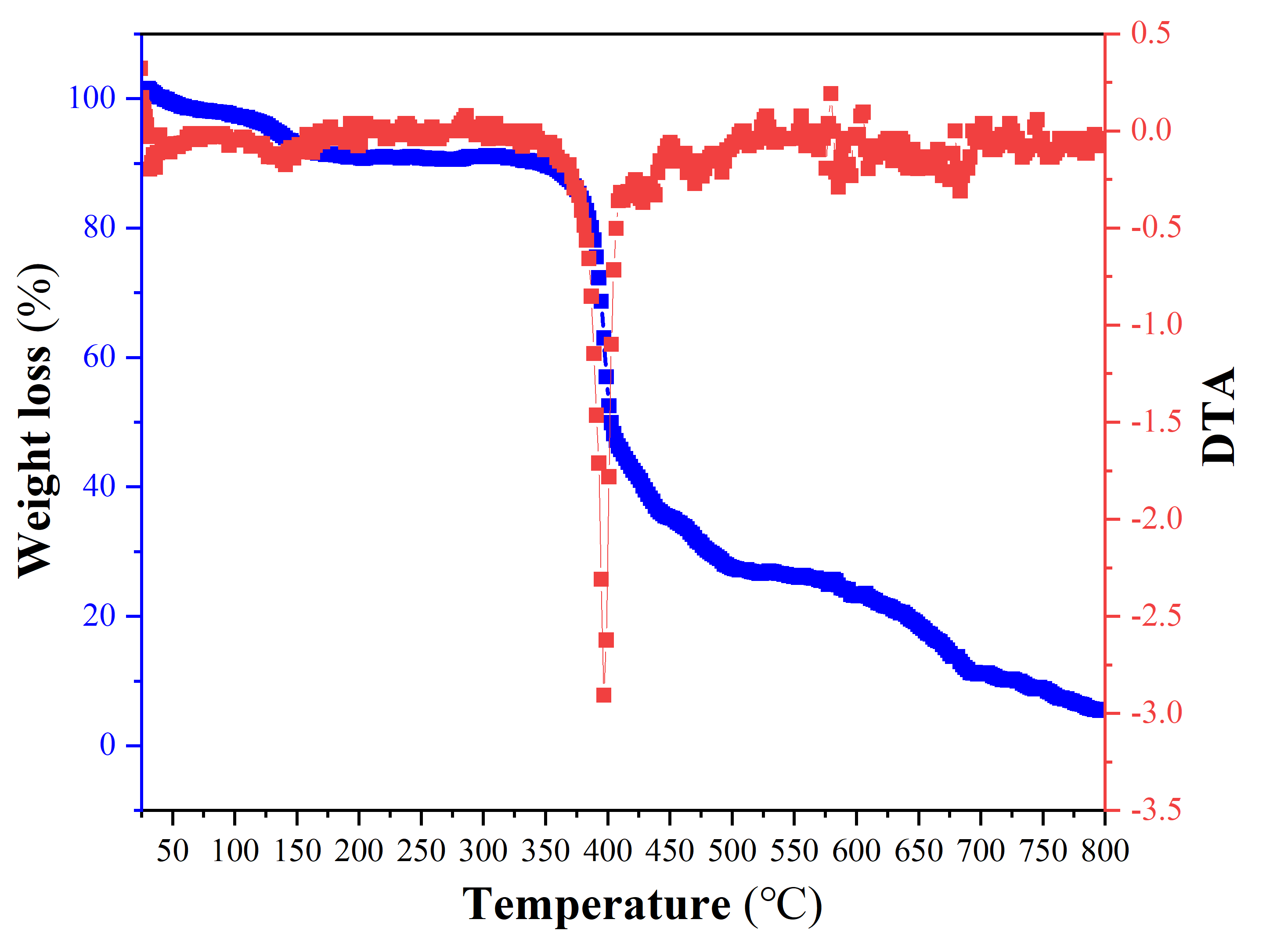


**Figure S1** Thermogravimetric analysis of **CPOSs-NXU-1**


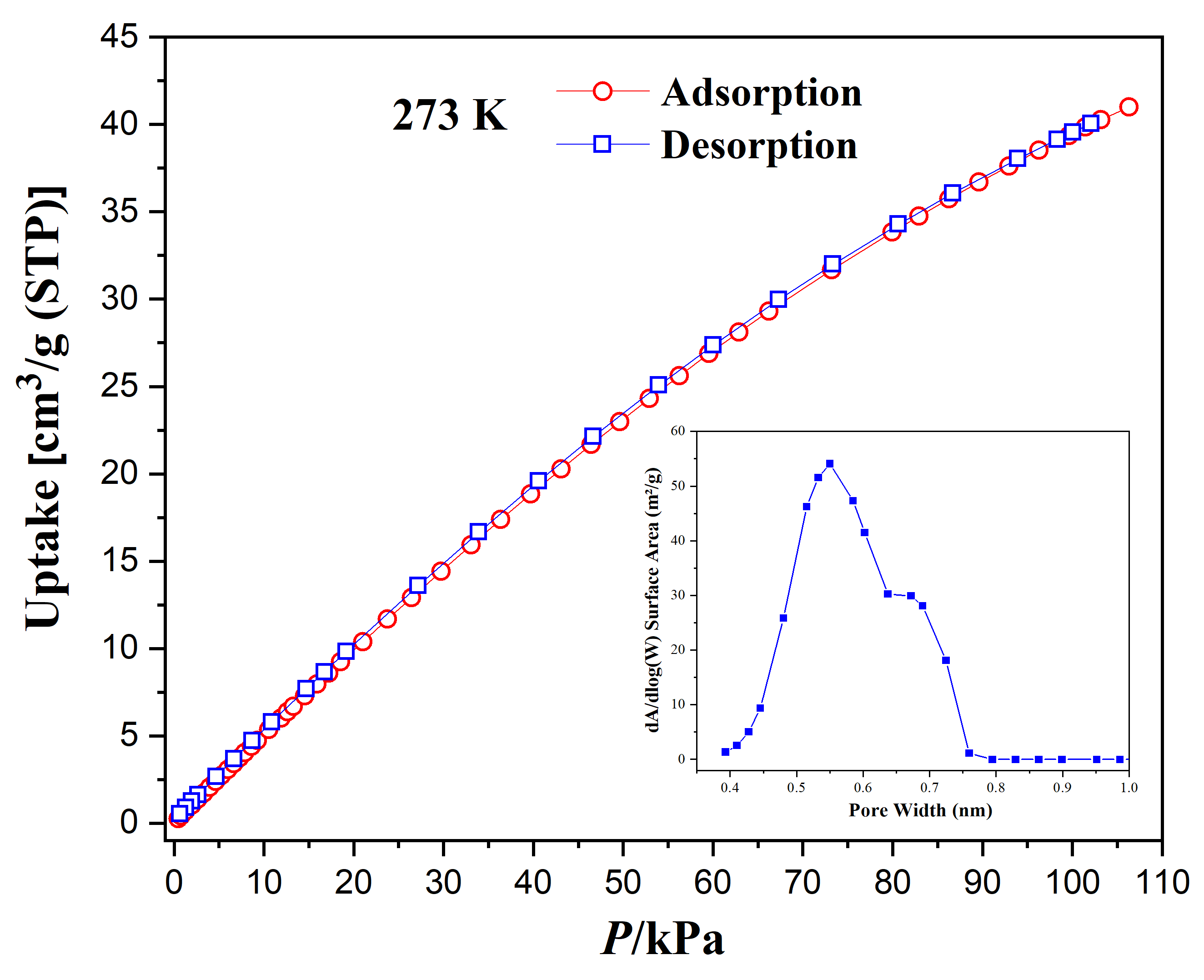


**Figure S2** CO_2_ adsorption and pore width distribution (inset) of **CPOSs-NXU-1** at 273 K.


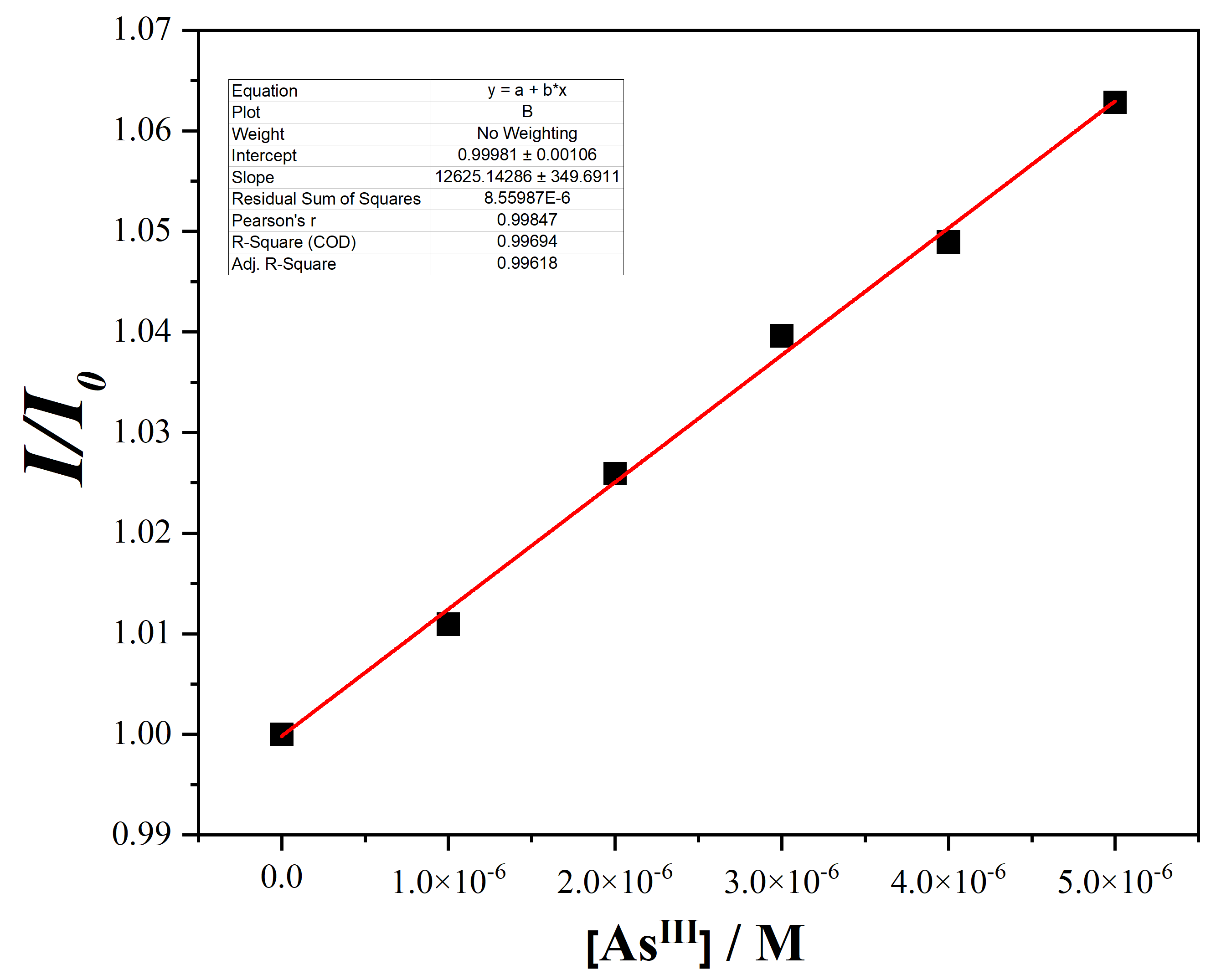


**Figure S3** Linear relationship of **CPOSs-NXU-1** to As^III^ ions in water at 0.0-5.0 μM.


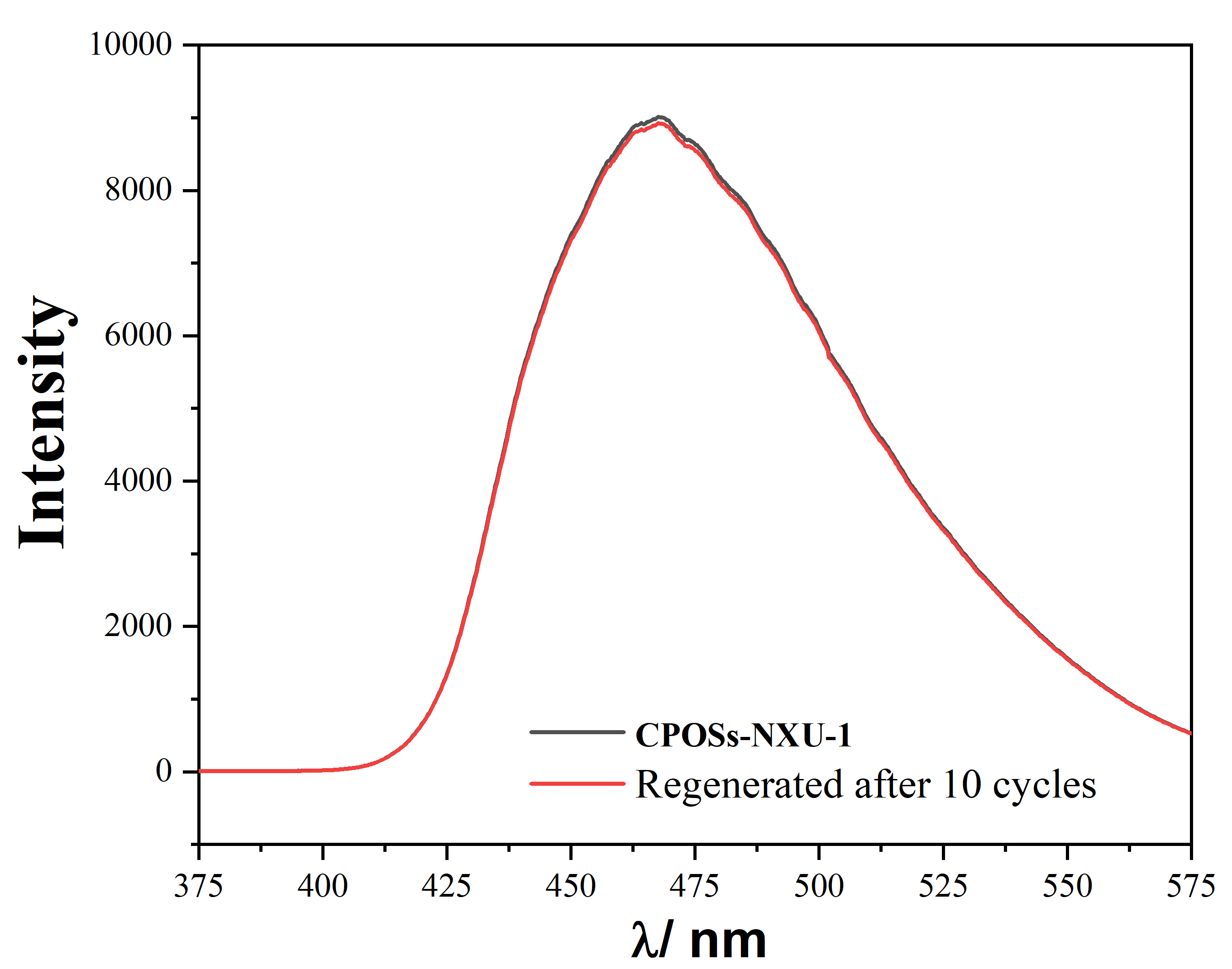


**Figure S4** Luminescence intensity of **CPOSs-NXU-1** and regenerated **CPOSs-NXU-1** after 10 sensing cycles.


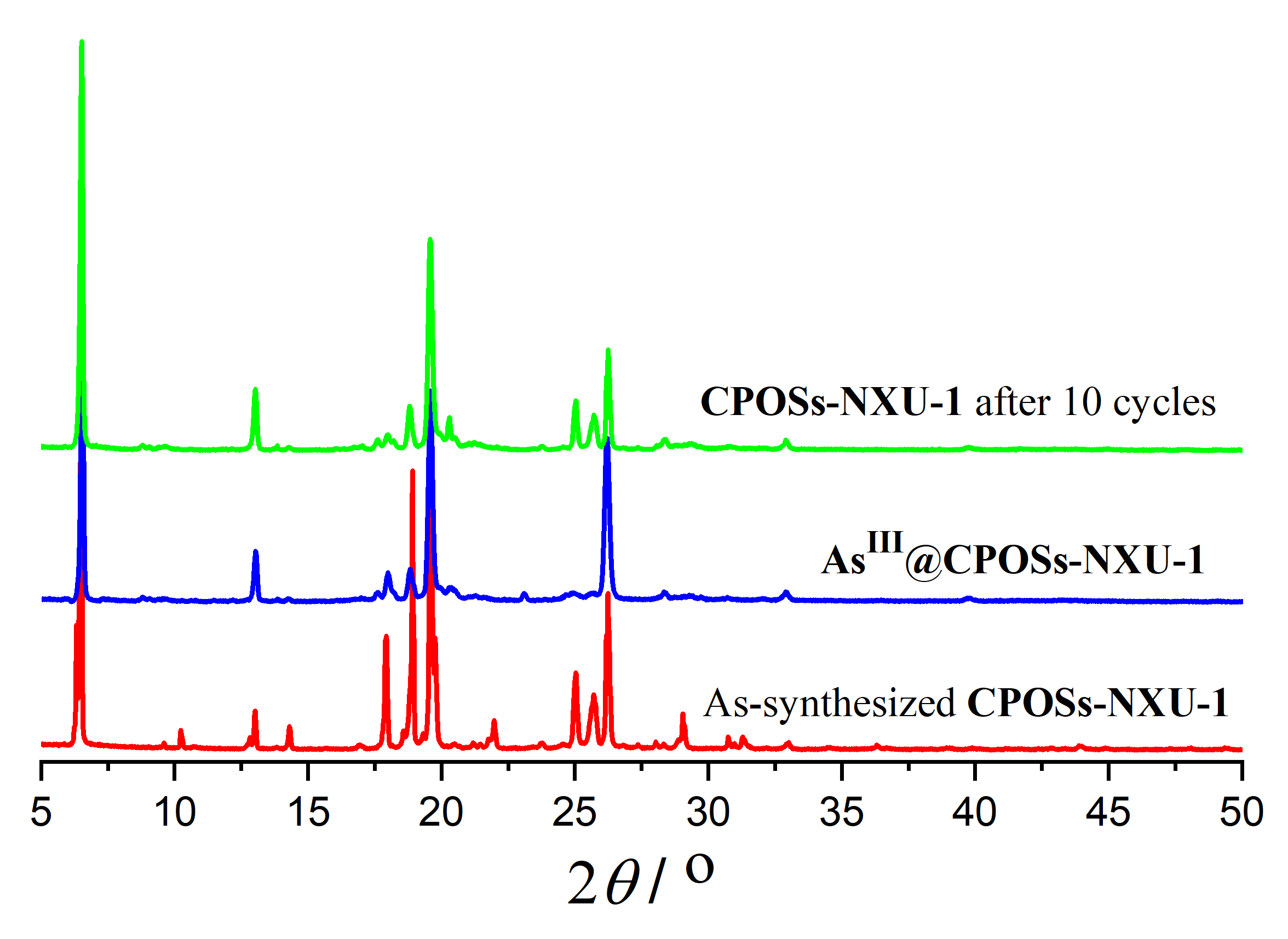


**Figure S5** PXRD of **CPOSs-NXU-1**, **As^III^@CPOSS-NXU-1**, and regenerated **CPOSs-NXU-1** after 10 sensing cycles.
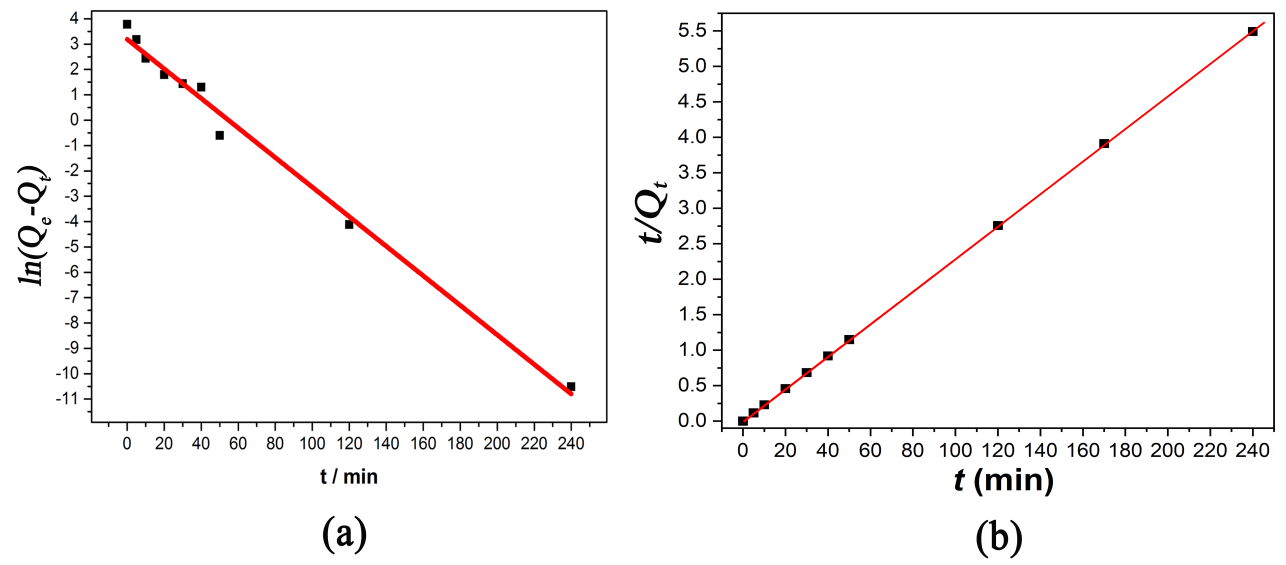


**Figure S6** The quasi-primary (a) and quasi-secondary (b) kinetic model fitting.


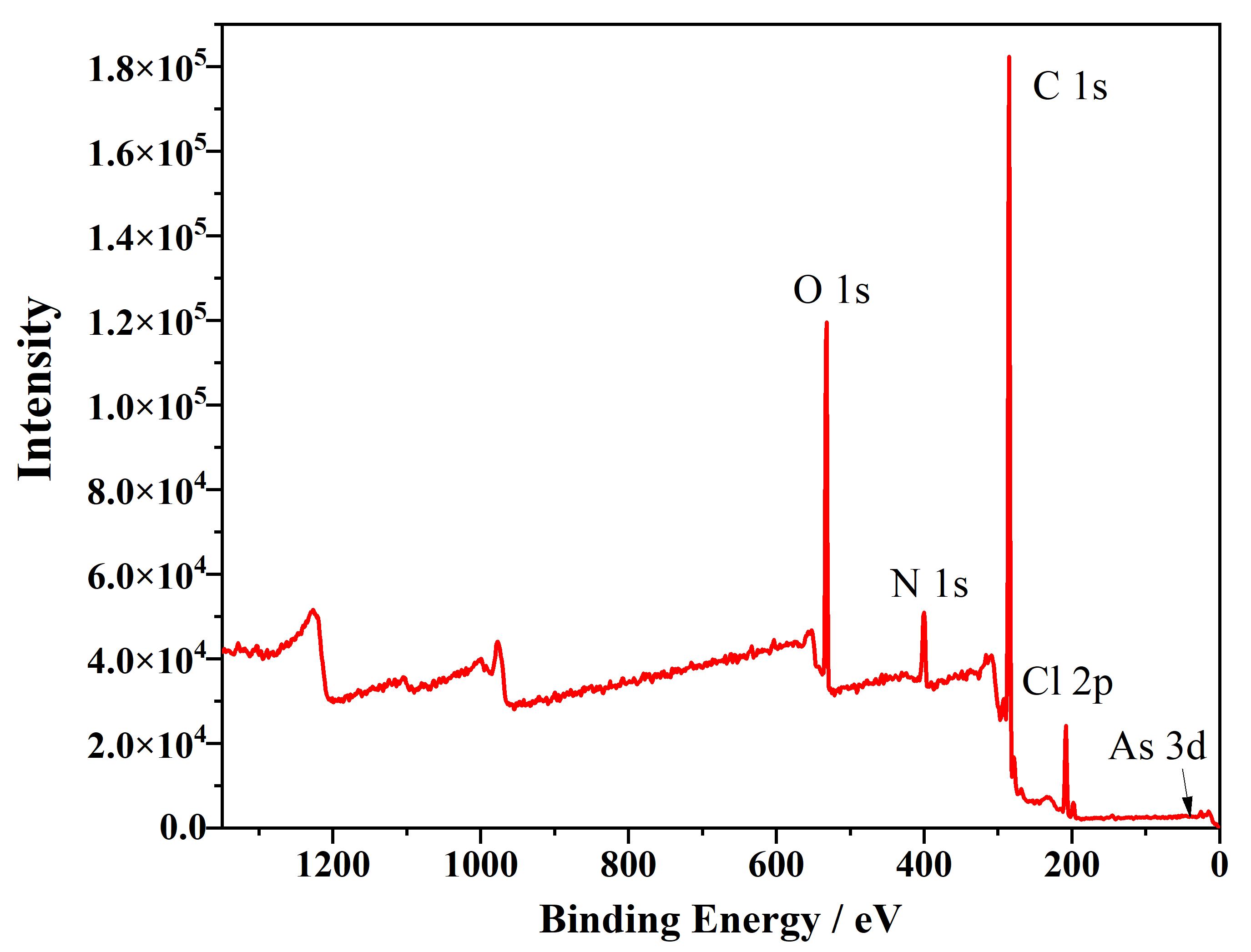


**Figure S7** The XPS spectra of **As^III^@ CPOSs-NXU-1**.


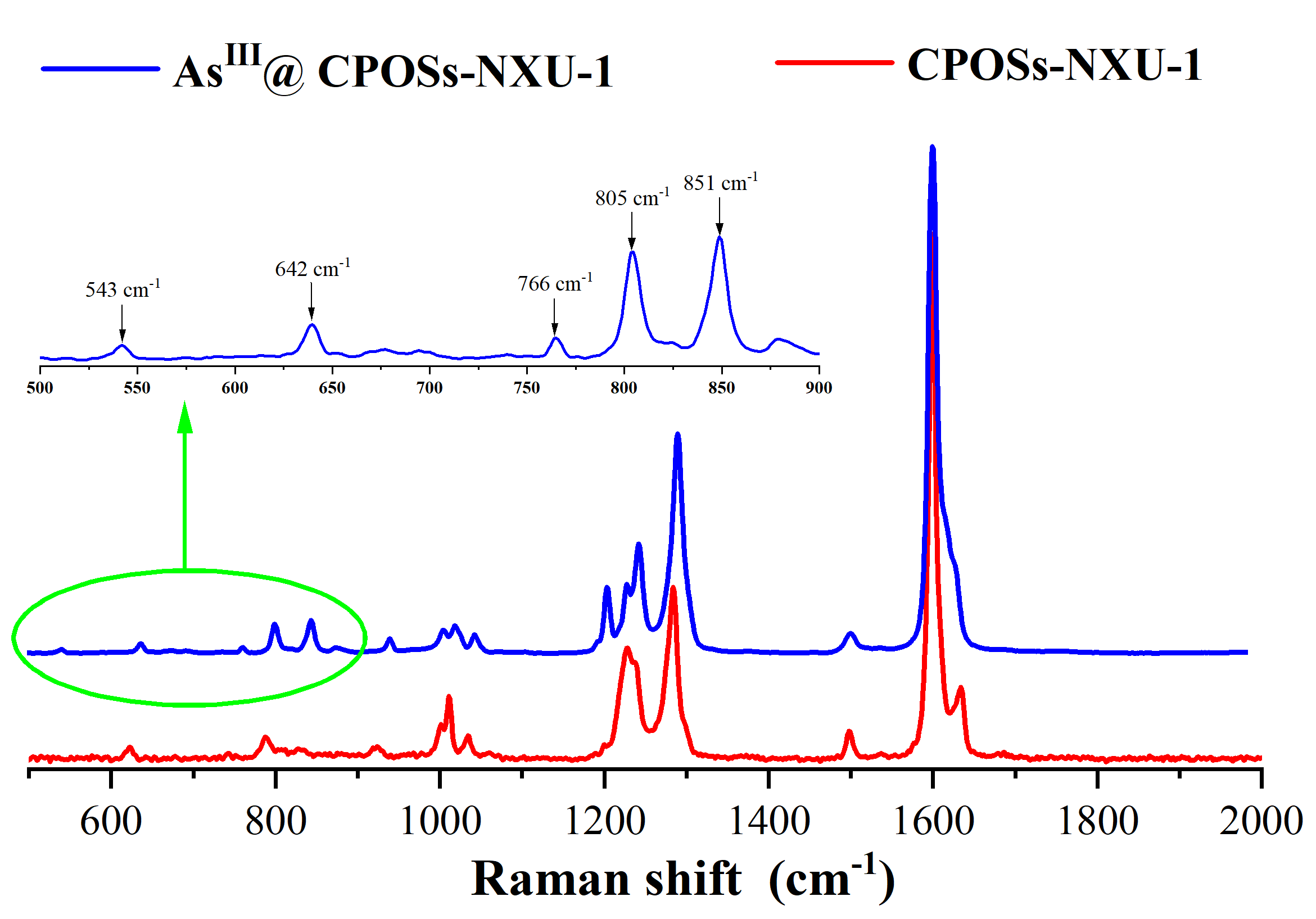


**Figure S8** Raman spectra of **CPOSs-NXU-1** and **As^III^@ CPOSs-NXU-1**.

**
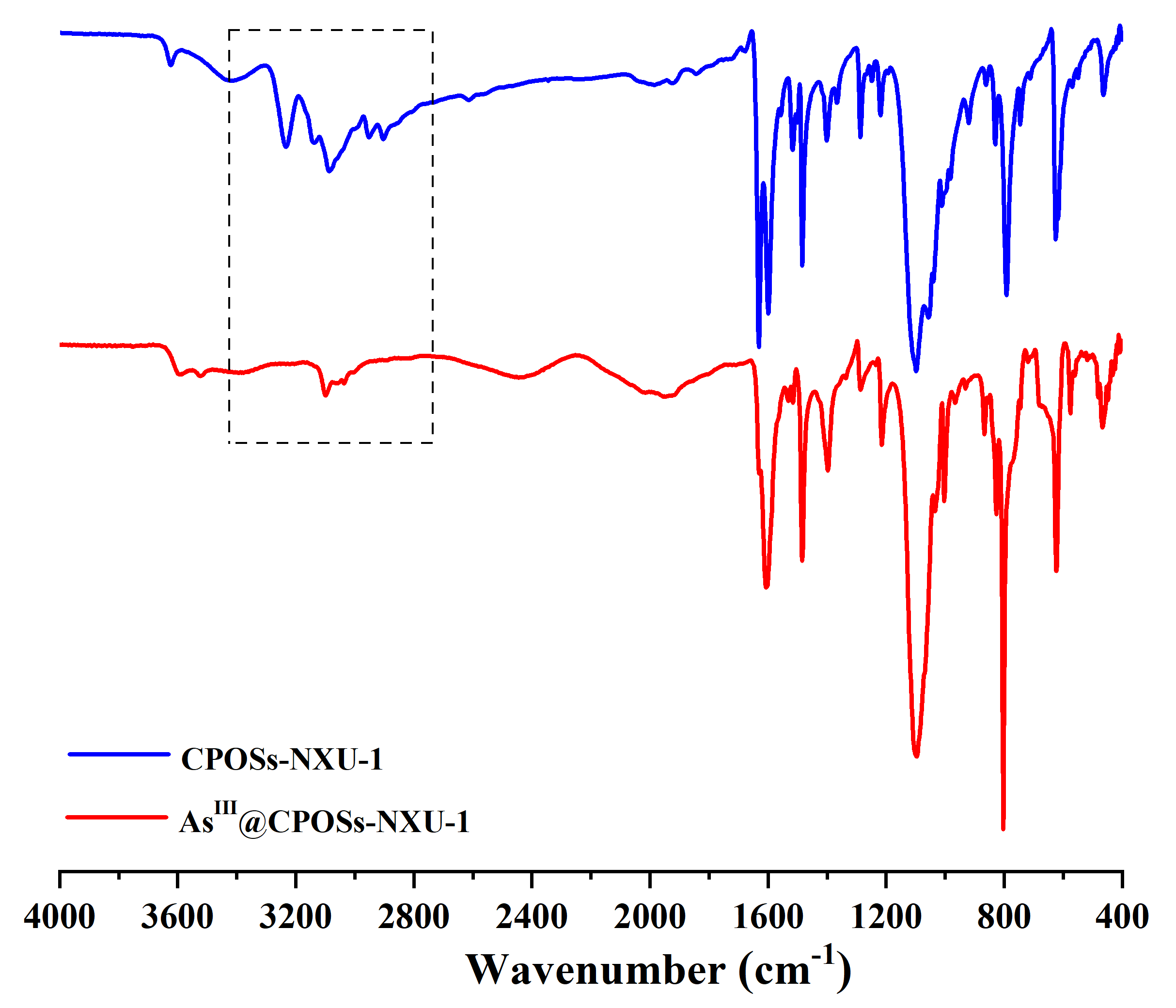
**

**Figure S9** IR spectra of **CPOSs-NXU-1** and **As^III^@ CPOSs-NXU-1**.

**Tables:**

**Table S1** Crystallographic and Refinement Parameters for **CPOSs-NXU-1**

| Complex | **CPOSs-NXU-1** |
| --- | --- |
| *Formula* | C_22_H_18_N_2_·2(ClO_4_) |
| *Formula Weight* | 509.28 |
| *Crystal System* | triclinic |
| *Space group* | ***P***‾1 |
| *a (Å)* | 4.9446(2) |
| *b (Å)* | 9.3499(3) |
| *c (Å)* | 13.8541(5) |
| *α (°)* | 76.654(2) |
| *β (°)* | 86.462(2) |
| *γ (°)* | 86.451(2) |
| *V (Å3 )* | 621.26(4) |
| *Z* | 1 |
| *D_calc_ (g·cm^−3^ )* | 1.361 |
| *F(000)* | 262 |
| *R_int_* | 0.07 |
| *GOF (S)* | 1.11 |
| *R_1_ [I > 2σ(I)]* | 0.0672 |
| *wR_2_ (all data)* | 0.1999 |

**Table S2** Isotherm parameters of As^III^ adsorption by **CPOSs-NXU-1**

| Isotherm model | Constants |  | Temp. (K) |  |
| --- | --- | --- | --- | --- |
|  |  | 273 | 293 | 303 |
| Baudu | *Q_m_* (mg/g) | 748.99±144.99 | 480.54±54.30 | 451.01±37.18 |
|  | *K*  *n* | 0.08675  0.07107 | 0.1121  0.1368 | 0.08877  0.2688 |
|  | *R^2^* | 0.9970 | 0.9951 | 0.9953 |

**Table S3** Kinetic parameters of As^III^ adsorption by **CPOSs-NXU-1**

| Kinetic Model | Parameters |
| --- | --- |
| Pseudo-ﬁrst-order model | *Q_e_* (exp) (mg/g) *Q_e_*(cal) (mg/g)  *k_1_* (min^-1^) *R^2^*  43.71 35.44 0.05820 0.98858 |
| Pseudo-second-order model | *Q_e_* (exp) (mg/g) *Q_e_*(cal) (mg/g) *k_2_* (g/(mg🞘min) ) *R^2^*  43.71 43.63 0.02292 0.99999 |
