# Supplementary material for: Integrated Solution for As(III) Contamination in Water Based on Crystalline Porous Organic Salts
Source: Adv Sci (Weinh). 2024 Jun 25;11(32):2403539. doi: 10.1002/advs.202403539 (PMC11348186; doi:10.1002/advs.202403539)

checkCIF/PLATON report

Structure factors have been supplied for datablock(s) cu_0201_2_0m_sq

THIS REPORT IS FOR GUIDANCE ONLY. IF USED AS PART OF A REVIEW PROCEDURE FOR PUBLICATION, IT SHOULD NOT REPLACE THE EXPERTISE OF AN EXPERIENCED CRYSTALLOGRAPHIC REFEREE.

No syntax errors found. CIF dictionary Interpreting this report

Datablock: cu_0201_2_0m_sq

Bond precision:	C-C = 0.0050 A		Wavelength=1.54178
Cell:	a=4.9446(2)	b=9.3499(3)	c=13.8541(5)
	alpha=76.654(2)	beta=86.462(2)	gamma=86.451(2)
Temperature:	150 K		

	Calculated	Reported
Volume	621.26(4)	621.26(4)
Space group	P -1	P -1
Hall group	-P 1	-P 1
Moiety formula	C22 H18 N2, 2(Cl O4) [+ solvent]	C22 H18 N2, 2(Cl O4)
Sum formula	C22 H18 Cl2 N2 O8 [+ solvent]	C22 H18 Cl2 N2 O8
Mr	509.28	509.28
Dx,g cm-3	1.361	1.361
Z	1	1
Mu (mm-1)	2.776	2.776
F000	262.0	262.0
F000'	263.56	
h,k,lmax		5,11,16
Nref		2255
Tmin,Tmax	0.659,0.717	0.424,0.754
Tmin'	0.532	

Correction method= # Reported T Limits: Tmin=0.424 Tmax=0.754
AbsCorr = MULTI-SCAN

Data completeness= Theta(max)= 68.225

R(reflections)= 0.0672(2072)   wR2(reflections)= 0.1999(2255)

S = 1.105   Npar= 192

The following ALERTS were generated. Each ALERT has the format
test-name_ALERT_alert-type_alert-level.
Click on the hyperlinks for more details of the test.

Alert level C

```
PLAT340_ALERT_3_C Low Bond Precision on  C-C Bonds ...............      0.005 Ang.
PLAT790_ALERT_4_C Centre of Gravity not Within Unit Cell: Resd.  #          1 Note
              C22 H18 N2
PLAT911_ALERT_3_C Missing FCF Refl Between Thmin & STh/L=    0.600          8 Report
                0  2  1,   1 -1  2,   2  2  2,   0  3  2,   1  1  3,   1 11  3,
                1  1  4,   1  9  6,
```

Alert level G

```
PLAT007_ALERT_5_G Number of Unrefined Donor-H Atoms .............           1 Report
              H1
PLAT072_ALERT_2_G SHELXL First  Parameter in WGHT  Unusually Large       0.12 Report
PLAT154_ALERT_1_G The s.u.'s on the Cell Angles are Equal ..(Note)      0.002 Degree
PLAT244_ALERT_4_G Low    'Solvent' Ueq as Compared to Neighbors of        Cl1 Check
PLAT301_ALERT_3_G Main Residue  Disorder .............(Resd  1  )        33% Note
PLAT410_ALERT_2_G Short Intra H...H Contact  H2       ..H7       .       2.09 Ang.
                                                      x,y,z  =     1_555 Check
PLAT410_ALERT_2_G Short Intra H...H Contact  H2       ..H7A      .       2.13 Ang.
                                                      x,y,z  =     1_555 Check
PLAT410_ALERT_2_G Short Intra H...H Contact  H4       ..H11      .       2.06 Ang.
                                                      x,y,z  =     1_555 Check
PLAT410_ALERT_2_G Short Intra H...H Contact  H4       ..H11A     .       2.03 Ang.
                                                      x,y,z  =     1_555 Check
PLAT605_ALERT_4_G Largest Solvent Accessible VOID in the Structure         91 A**3
PLAT869_ALERT_4_G ALERTS Related to the Use of SQUEEZE  Suppressed          ! Info
PLAT883_ALERT_1_G No Info/Value for _atom_sites_solution_primary .     Please Do !
PLAT913_ALERT_3_G Missing # of Very Strong Reflections in FCF ....          1 Note
                1 -1  2,
PLAT933_ALERT_2_G Number of HKL-OMIT Records in Embedded .res File          1 Note
                1  1  4,
PLAT978_ALERT_2_G Number C-C Bonds with Positive Residual Density.          1 Info
PLAT992_ALERT_5_G Repd & Actual _reflns_number_gt Values Differ by          2 Check
```

---

0 **ALERT level A** = Most likely a serious problem - resolve or explain
0 **ALERT level B** = A potentially serious problem, consider carefully
3 **ALERT level C** = Check. Ensure it is not caused by an omission or oversight
16 **ALERT level G** = General information/check it is not something unexpected

2 ALERT type 1 CIF construction/syntax error, inconsistent or missing data
7 ALERT type 2 Indicator that the structure model may be wrong or deficient
4 ALERT type 3 Indicator that the structure quality may be low
4 ALERT type 4 Improvement, methodology, query or suggestion
2 ALERT type 5 Informative message, check

Validation response form

Please find below a validation response form (VRF) that can be filled in and pasted into your CIF.

```
# start Validation Reply Form
_vrf_PLAT340_cu_0201_2_0m_sq
;
PROBLEM: Low Bond Precision on  C-C Bonds ...............      0.005 Ang.
RESPONSE: ...
;
_vrf_PLAT790_cu_0201_2_0m_sq
;
PROBLEM: Centre of Gravity not Within Unit Cell: Resd.  #          1 Note
RESPONSE: ...
;
_vrf_PLAT911_cu_0201_2_0m_sq
;
PROBLEM: Missing FCF Refl Between Thmin & STh/L=    0.600          8 Report
RESPONSE: ...
;
# end Validation Reply Form
```

It is advisable to attempt to resolve as many as possible of the alerts in all categories. Often the minor alerts point to easily fixed oversights, errors and omissions in your CIF or refinement strategy, so attention to these fine details can be worthwhile. In order to resolve some of the more serious problems it may be necessary to carry out additional measurements or structure refinements. However, the purpose of your study may justify the reported deviations and the more serious of these should normally be commented upon in the discussion or experimental section of a paper or in the "special_details" fields of the CIF. checkCIF was carefully designed to identify outliers and unusual parameters, but every test has its limitations and alerts that are not important in a particular case may appear. Conversely, the absence of alerts does not guarantee there are no aspects of the results needing attention. It is up to the individual to critically assess their own results and, if necessary, seek expert advice.

Publication of your CIF in IUCr journals

A basic structural check has been run on your CIF. These basic checks will be run on all CIFs submitted for publication in IUCr journals (*Acta Crystallographica*, *Journal of Applied Crystallography*, *Journal of Synchrotron Radiation*); however, if you intend to submit to *Acta Crystallographica Section C* or *E* or *IUCrData*, you should make sure that full publication checks are run on the final version of your CIF prior to submission.

Publication of your CIF in other journals

Please refer to the *Notes for Authors* of the relevant journal for any special instructions relating to CIF submission.

PLATON version of 14/11/2023; check.def file version of 14/09/2023

Datablock cu_0201_2_0m_sq - ellipsoid plot

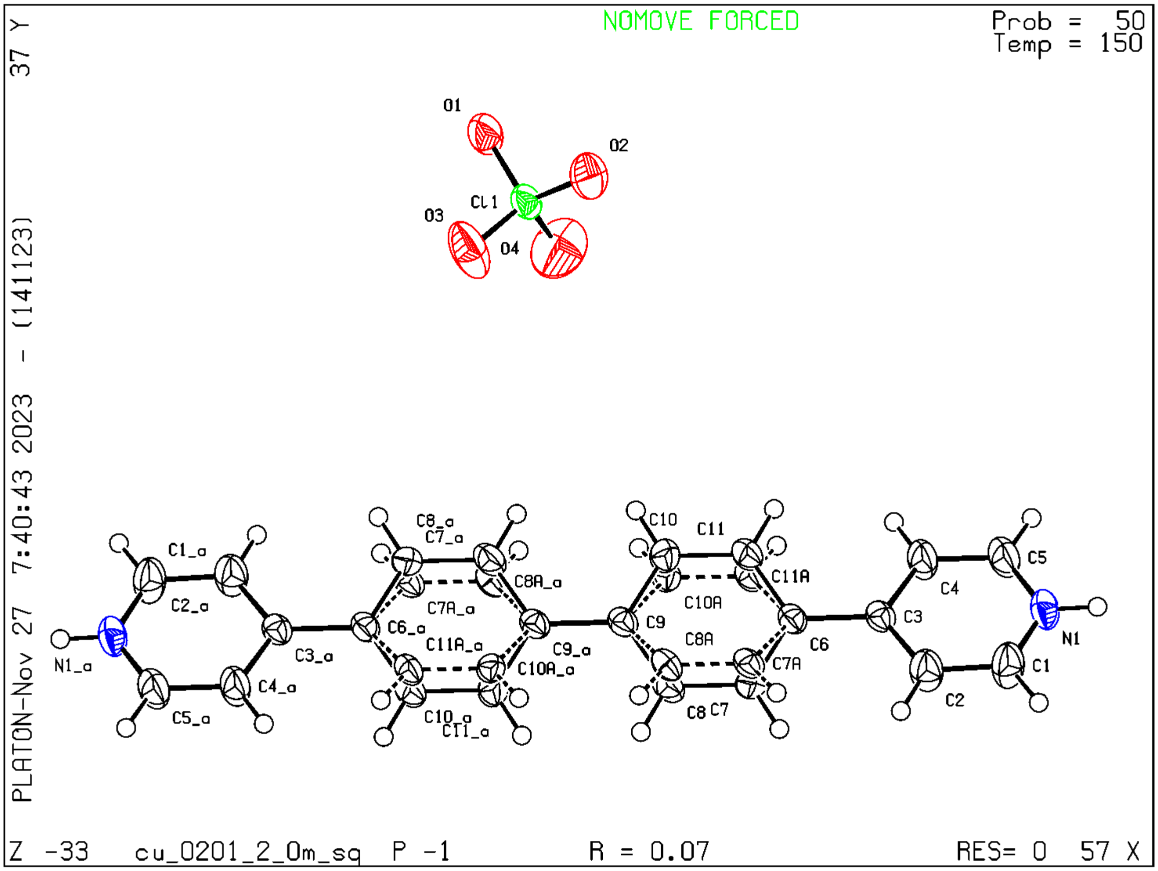

Supplement: Supplementary file 2 — Supporting Information [file ADVS-11-2403539-s001.zip › Crystaldata/CPOSs-NXU-1 cifreport.pdf]
